# Effect of the fragrance inhalation of essential oil from *Asarum heterotropoides* on depression-like behaviors in mice

**DOI:** 10.1186/s12906-015-0571-1

**Published:** 2015-03-06

**Authors:** Hyun-Jung Park, Eun-Ju Lim, Rong Jie Zhao, Sa Rang Oh, Ji Wook Jung, Eun-Mi Ahn, Eun Sook Lee, Jin Suk Koo, Hee Young Kim, Suchan Chang, Hyun Soo Shim, Kwang Joong Kim, Young Seob Gwak, Chae Ha Yang

**Affiliations:** Bringham Young University, Provo, Utah 84602 USA; College of Korean Medicine, Daegu Hanny University, Daegu, 706-828 South Korea; Mudanjiang Medical University, Mudanjiang, 157011 China; Andong National University, Andong, Gyeongsangbuk-do 760-749 South Korea; Kyung Hee University, Seoul, 130-701 South Korea

**Keywords:** Essential oil, *Asarum heterotropoides*, Depression, Inhalation

## Abstract

**Background:**

Psychological stressors may cause affective disorders, such as depression and anxiety, by altering expressions of corticotropin releasing factor (CRF), serotonin (5-HT), and tyrosine hydroxylase (TH) in the brain. This study investigated the effects of essential oil from *Asarum heterotropoides* (EOAH) on depression-like behaviors and brain expressions of CRF, 5-HT, and TH in mice challenged with stress.

**Methods:**

Male ICR mice received fragrance inhalation of EOAH (0.25, 0.5, 1.0, and 2.0 g) for 3 h in the special cage capped with a filter paper before start of the forced swimming test (FST) and tail suspension test (TST). The duration of immobility was measured for the determination of depression-like behavior in the FST and TST. The selective serotonin reuptake inhibitor fluoxetine as positive control was administered at a dose of 15 mg/kg (i.p.) 30 min before start of behavioral testing. Immunoreactivities of CRF, 5-HT, and TH in the brain were also measured using separate groups of mice subjected to the FST.

**Results:**

EOAH at higher doses (1.0 and 2.0 g) reduced immobility time in the FST and TST. In addition, EOAH at a dose of 1.0 g significantly reduced the expected increases in the expression of CRF positive neurons in the paraventricular nucleus and the expression of TH positive neurons in the locus coeruleus, and the expected decreases of the 5-HT positive neurons in the dorsal raphe nucleus.

**Conclusion:**

These results provide strong evidence that EOAH effectively inhibits depression-like behavioral responses, brain CRF and TH expression increases, and brain 5-HT expression decreases in mice challenged with stress.

## Background

Physical and psychological stressors are thought to cause affective disorders including depression and anxiety [1,2]. The neurobiological substrate for depression-like behaviors induced by stress is believed to involve corticotropinergic neurons in the paraventricular nucleus, noradrenergic neurons in the locus coeruleus, and serotonergic system in the dorsal raphe nucleus [3,4]. The involvement of these substrates is supported by the report that selective serotonin reuptake inhibitor (SSRI), serotonin-norepinephrine reuptake inhibitor (SNRI), norepinephrine-dopamine reuptake inhibitor, and inhibitor of hypothalamic-pituitary-adrenal (HPA) axis are used for the treatment of depression [5]. However, the clinical satisfaction is still under debating due to unwanted side effects and genetic variation. Therefore, the alternative medication is necessary to provide effective approaches for reducing the depression.

Studies in laboratory animals and humans have demonstrated that inhalation of essential oils can produce antidepressant and anxiolytic effects exposed to stress by modulating the central nervous system. For examples, inhalation of lemon oil reduced anxiogenic and depressant effects in an elevated plus-maze test and a forced swimming test (FST) by modulating serotonergic and dopaminergic pathways in mice brain [6]. In addition, lavender oil inhalation decreased anxiety and depression-like behaviors of rats in an elevated plus-maze test and a FST [7]. Lavender oil inhalation is also effective in reducing stress-related symptoms in nurses [8].

*Asarum heterotropoides Fr. Mandshuricum* has been shown to be effective for reducing anxiety and for the relief of pain [9,10]. Furthermore, biological and pharmacological studies have demonstrated that *Asarum heterotropoides* can produce wide spectrums of action including anti-inflammatory effect [11,12] and anti-allergic [13] and antioxidant activity [14].

The present study was carried out to investigate the effects of inhalation of essential oil from *Asarum heteropoides* (EOAH) on depression–like behaviors in the FST and tail suspension test (TST). A role for brain corticotropin releasing factor (CRF), serotonin (5-HT), and tyrosine hydroxylase (TH) expression in EOAH effects is also explored.

## Methods

### Animals

Male mice of ICR strain (Orient Inc., South Korea) at 5 weeks after the birth were housed under a controlled temperature (23 ± 2°C) and humidity (50 ± 10%) with a 12 h light–dark cycle (lights on at 8:00 h). Food and water were made available *ad libitum* for the study. All animal experiments were carried out in accordance with the animal care guidelines of the National Institute of Health (NIH) and approved by the Institutional Animal Care and Use Committee of Daegu Hanny University.

### Preparation of EOAH and chemical analysis

The dried and chipped roots of *Asarum heterotropoides* (1.2 kg) were purchased from Daegu Yangnyeongsi (a traditional herb market) in Daegu, South Korea. The pulverized *Asarum heterotropoides* were used to extract essential oil at the room temperature with *n*-hexane (5 L x 2) for 24 h. The extracts (EOAH) were filtered and evaporated in the vacuum at 40°C to remove *n*-hexane. The final concentration of essential oil was 22.5 g. The specimen of this plant material (RCBROM-AEM2012) is deposited at the Research Center for Biomedical Resources of Oriental Medicine, Daegu Haany University. EOAH was analyzed for the determination of chemical composition using GC-MS (Agilent Technologies 7890A/5975C insert XL Mass Selective detector) with injection volume 1 μl (Column: DB-5MS; 250 μm i.d.; 30 m length; 0.25 μm film thickness; split ratio 1:50. Carrier: Helium. Injector 250°C, Detector 280°C, Column 70°C, 1 min; 4°C/min to 300°C for 20 min). The identification of EOAH was carried out by comparing their relative retention time and mass spectra to those generated by chromatographic analysis of standards and W9N08.L library and literature.

### Inhalation of EOAH

To evaluate the effects of EOAH on depression-like behaviors, the mice were randomly divided into the following 5 groups: Control (*n* = 5); EOAH 0.25 g (*n* = 5); EOAH 0.5 g (*n* = 5); EOAH 1.0 g (*n* = 5); EOAH 2.0 g (*n* = 5). Mice weighing between 25 and 30 g at the start of the experiment were used. The mice were pretreated with inhalation of EOAH (0.25, 0.5, 1.0, and 2.0 g) or saline (control group). The inhalation dose of essential oil was chosen based on previous studies [15,16]. To induce saturation of the fragrance by EOAH in the transparent special cage (W 26 X L 22 X H 20 cm), EOAH put 30 min before the individual inhalation of EOAH for 3 h in the special cage under standardized condition (room temperature: 23 ± 2°C, relative humidity: 50 ± 10%). The special cage was capped with a filter paper, which allowed minimum breathing air to pass. In order to vaporize essential oil efficiently, EOAH was put in uncapped eppendorf tube set on the upper side of an inhalation cage. After inhalation of EOAH, no mice have shown abnormal behaviors including enhanced locomotion, loss of body postures, sweats, head turns and vocalization. The selective serotonin reuptake inhibitor fluoxetine (Sigma-Aldrich, St. Louis, MO, U.S.A.) was dissolved in saline. Mice were treated with 15 mg/kg of fluoxetine (*n* = 7) as positive control or saline (control group, *n* = 7) by intraperitoneal injection 30 min before the start of behavioral testing. An injection volume was 0.3 ml/30 g body weight.

### FST and TST

Following inhalation of EOAH, the mice were subjected to the FST and TST, respectively. The duration of immobility induced by forced swimming was measured as described previously with slight modification [17]. The mice were individually exposed to forced swimming in a transparent acrylic cylinder (25 cm in height and 10 cm in diameter) containing 20 cm height of water at 23°C. The immobility was defined as floating motionless in the water [18]. The duration of immobility was measured during the last 4 min of total 6 min test using a video based Ethovision System (Noldus, Wageningen, Netherlands). TST was carried out according to the method described previously with a slight modification [19]. In brief, the mice were individually hung 5 cm above the floor in clear black acrylic boxes (30 cm × 30 cm × 50 cm) by tail attachment using an adhesive tape to a hook. The mice were individually allowed to hang for 6 min and the duration of immobility was recorded during the last 4 min of total 6 min test. The immobility was defined as absence of body movements [19].

### Immunohistochemistry

The expressions of 5-HT, TH, and CRF were measured using different groups of mice. Mice were divided 4 groups: Normal (*n* = 8); Control (*n* = 9); EOAH 0.5 g (*n* = 9); EOAH 1.0 g (*n* = 9). At the end of the FST, mice were sacrificed and brains were prepared to measure the expressions of 5-HT, TH, and CRF, respectively. Mice in normal group were not allowed to have the FST. Immunohistological study began with transcardial perfusion of heparinized phosphate-buffered saline (PBS; pH 7.4) for 30 s followed by perfusion of 4% paraformaldehyde in 0.1 M phosphate buffered saline (pH 7.4) for 10–15 min. Brains were removed and allowed to stand in the same fixative by overnight, followed by cryoprotected in 30% sucrose solution in PBS. The individual brain was embedded in OCT compound and serially sectioned on a cryostat (Leica, Nussloch, Germany) at 30 μm thickness by the coronal plane, and then collected in PBS using free floating methods. The individual primary antibody includes anti-5HT (rabbit monoclonal, 1:200; Abcam, Cambridge, U.S.A.), anti-CRF (rabbit monoclonal, 1:200; Abcam, Cambridge, U.S.A.), and anti-TH (rabbit monoclonal, 1:2000; Abcam, Cambridge, U.S.A.) was incubated in a cocktail solution (0.3% PBST, 2% blocking serum and 0.001% keyhole limpet hemocyanin) for 72 h at 4°C, respectively. After three time rinses in PBS, the sections were placed in Vectastain Elite ABC reagent (Vector laboratories, Burlingame, CA, U.S.A.) for 2 h at room temperature. Following a further rinsing in PBS, the tissue was developed using diaminobenzadine (Sigma-Aldrich, St. Louis, MO, U.S.A.) as the chromogen. The images of 5-HT-, CRF-, and TH-immunoreactive neurons were captured using a DP2-BSW imaging system (Olympus, CA, USA) and measured as described by others [20]. In brief, the grid was placed on the target area in the brain and the number of cells was counted at 100 x magnification using a microscope rectangle grid. The five sections were collected at the levels of the dorsal raphe nucleus, the paraventricular nucleus and the locus coeruleus in each brain for 5-HT, CRF and TH immunoreaction. The cells were counted and averaged from five sections.

### Statistical analysis

Statistical analysis of data was carried out using SPSS 15.0 for Windows. Depression-like behaviors and brain 5-HT-, TH-, and CRF-immunoreactivity were statistically analyzed by one-way ANOVA and *post-hoc* Newman-Keuls test to compare the experimental and control groups. An unpaired *t*-test was performed to determine statistical significance for fluoxetine versus saline comparison. The significance level was set at *p* < 0.05, *p* < 0.01, and *p* < 0.001.

## Results

### Major compounds of EOAH

The total 78 peaks were detected in EOAH. The main compounds in analyzed samples were methyl eugenol (22.58%), pentadecane (6.78%), 2,3,5-trimethoxytoluene (5.54%), 4-(chloromethyl) cyclohexene (3.36%), myristicine (3.27%), sesamin (3.24%), and kakoul (2.63%).

### The effect of EOAH on the depression-like behaviors

**Figure 1:**
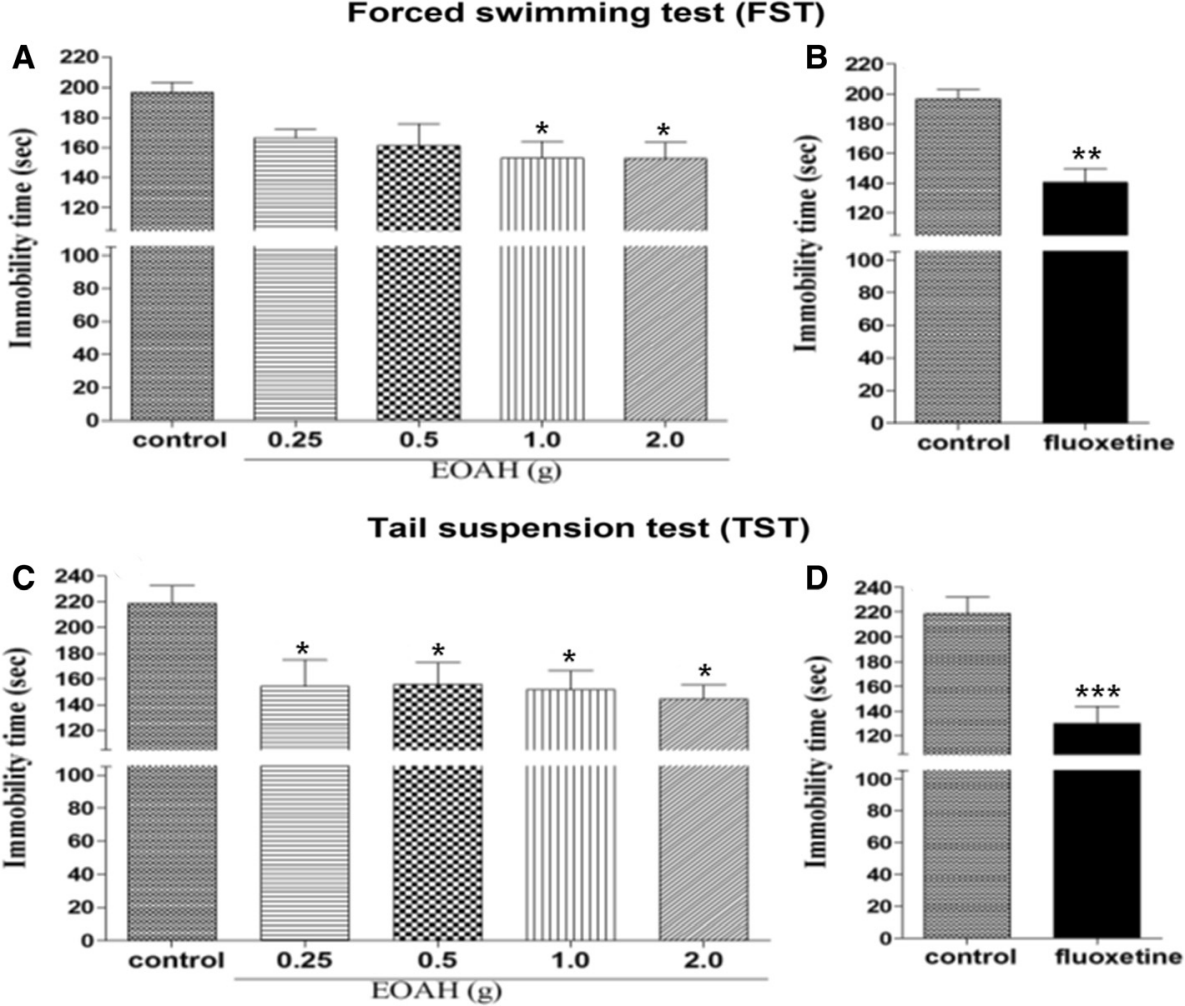
**Effects of inhalation of EOAH on the FST and TST in mice.** The mice were pretreated with inhalation of EOAH (0.25, 0.5, 1.0, and 2.0 g; EOAH groups) or saline (control group) prior to FST or TST. The graph **(A and C)** represents the immobility time during a 4 min period in all groups (*n* = 5 per each group). Data were analyzed by one-way ANOVA and *post-hoc* Newman-Keuls test. For comparative purpose, mice were administered with fluoxetine or saline. The graph **(B and D)** represents the immobility time during a 4 min period in all groups (*n* = 7 per each group). Data were analyzed by *t*-test. Each value represents the mean ± S.E.M. *p < 0.05 compared to the control group.

Inhalation of EOAH at higher doses (1.0 and 2.0 g) significantly reduced immobility time in the FST (Figure 1A). A one-way ANOVA identified a significant main effect (F_4,20_ = 3.19, *p* < 0.05). Post hoc tests revealed that mice receiving saline (control) had significantly greater immobility time than those treated with EOAH during the FST (*p* < 0.05). When mice were exposed to the TST, inhalation of EOAH (0.25, 0.5, 1.0, and 2.0 g) significantly decreased immobility time (Figure 1C). Interestingly, an antidepressant effect of EOAH at lower doses was more apparent in the TST. A one-way ANOVA identified a significant main effect (F_4,20_ = 3.63, *p* < 0.05). Post hoc tests revealed that mice receiving saline had significantly greater immobility time than those treated with EOAH during the TST (*p* < 0.05). Similarly, administration of fluoxetine markedly reduced immobility time at both the FST and TST compared to saline group (*p* < 0.01, *p* < 0.001, respectively, Figure 1B and D). This provides additional evidence that EOAH effectively reduces depression-like behaviors. In our study, EOAH at higher doses significantly suppressed depression-like behaviors in the FST. Thus, EOAH at a dose of 1.0 g was exposed to mice subjected to the FST in order to identify EOAH effects on brain levels of 5-HT, CRF, and TH.

### 5-HT-immunoreactive neurons in the dorsal raphe nucleus

**Figure 2:**
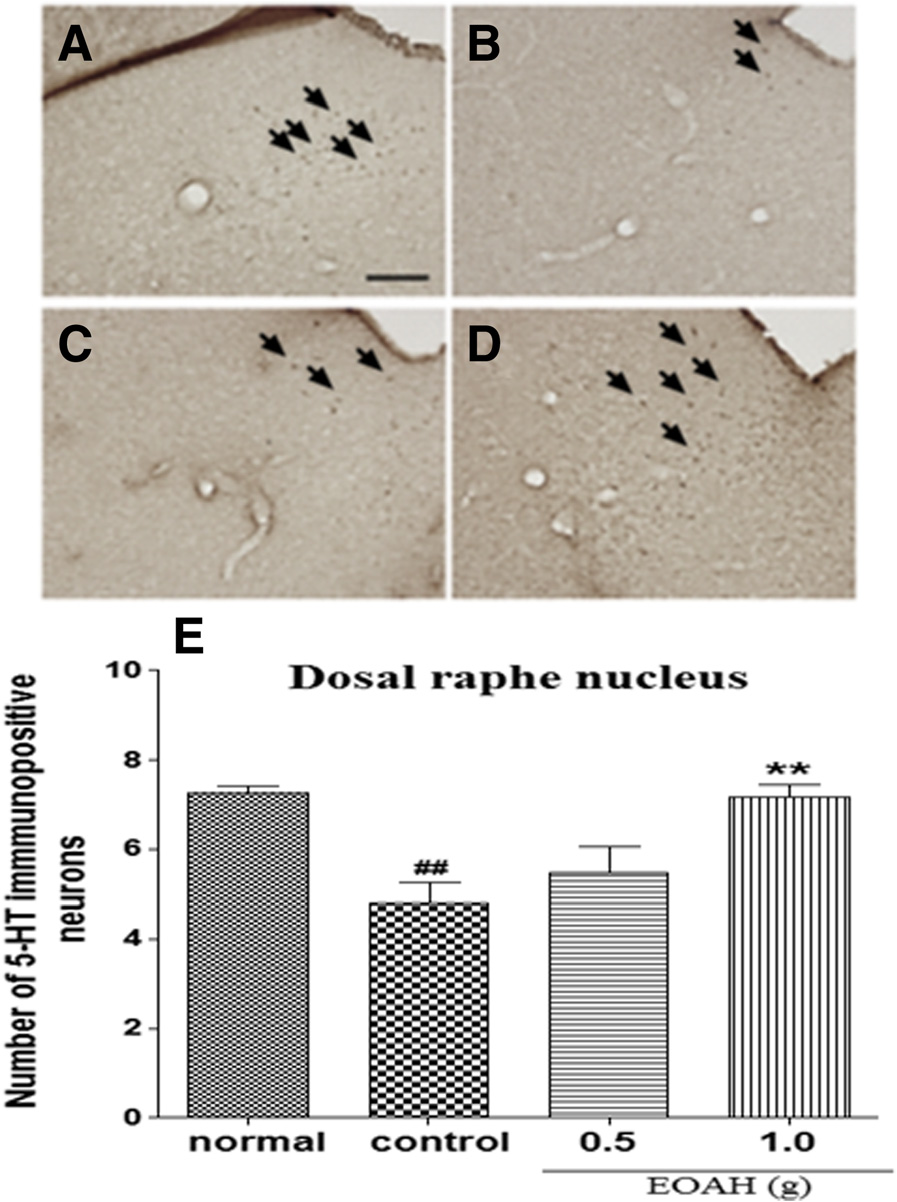
**Effect of inhalation of EOAH on 5-HT expression in the dorsal raphe nucleus in mice subjected to the FST.** Photographs represent the distribution of 5-HT-immunoreactive neurons in the dorsal raphe nucleus of normal (**A**, *n* = 8), control (**B**, *n* = 9), EOAH 0.5 g (**C**, *n* = 9) and EOAH 1.0 g (**D**, *n* = 9) groups. The number of 5-HT immunostained neurons among the groups (**E**) was analyzed by one-way ANOVA *post-hoc* Newman-Keuls test. Each value represents the mean ± S.E.M. ^##^ *p* < 0.01 compared to the normal group, **p < 0.01 compared to the control group. Sections were cut coronally at 30 μm and the scale bar represents 200 μm. Arrowheads indicate 5-HT immunopositive neurons.

Changes in the expression of 5-HT-immunoreactive neurons were evaluated in mice exposed to the FST following inhalation of EOAH (Figure 2A-D). As shown in Figure 2E, a much larger decrease in the mean number of 5-HT-immunoreactive neurons in the dorsal raphe nucleus was produced in mice exposed to the FST compared with normal mice. While the FST markedly decreased the expression of 5-HT in the dorsal raphe nucleus in mice receiving inhalation of saline (*p* < 0.01), inhalation of EOAH (1.0 g) increased 5-HT expression in the dorsal raphe nucleus. A one-way ANOVA analysis revealed a significant main effect of EOAH inhalation (F_3,31_ = 8.339, *p* < 0.001). Post-hoc comparisons indicated that there was a significant enhancement in the 5-HT activity in the dorsal raphe nucleus of the 1.0 g EOAH group compared with the control group (*p* < 0.01). Group 0.5 g EOAH mice tended to have greater 5-HT activity in the dorsal raphe nucleus than the control group, but these differences were not statistically significant.

### CRF-immunoreactive neurons in the paraventricular nucleus

**Figure 3:**
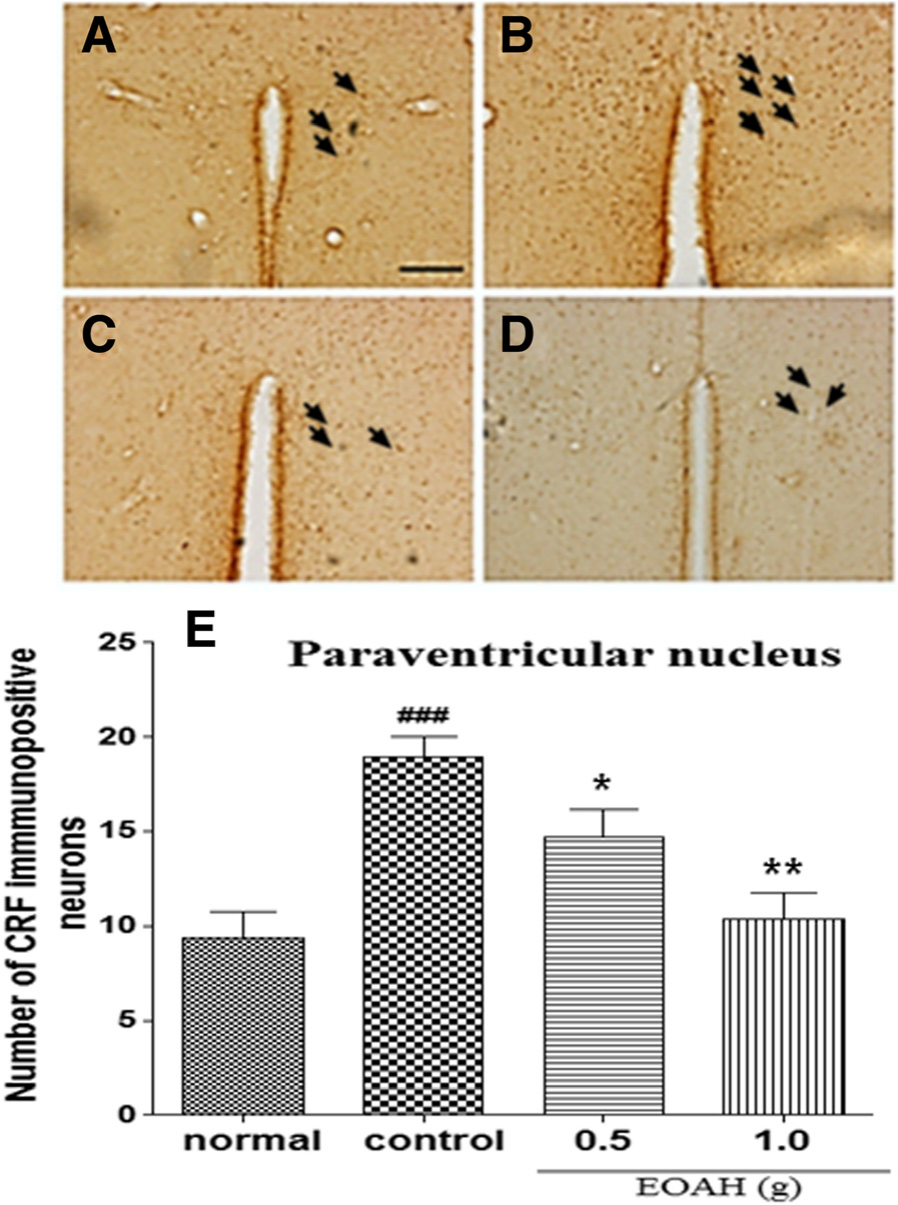
**Effect of inhalation of EOAH on CRF expression in the paraventricular nucleus in mice subjected to the FST.** Photographs represent the distribution of CRF-immunoreactive cells in the paraventricular nucleus of normal (**A**, *n* = 8), control (**B**, *n* = 9), EOAH 0.5 g (**C**, *n* = 9) and EOAH 1.0 g (**D**, *n* = 9) groups. The number of the CRF immunostained neurons among the groups (**E**) was analyzed by one-way ANOVA *post-hoc* Newman-Keuls test. Each value represents the mean ± S.E.M. ^###^ *p* < 0.001 compared to the normal group. *p < 0.05 and **p < 0.01 compared to the control group. Sections were cut coronally at 30 μm and the scale bar represents 200 μm. Arrowheads indicate CRF immunopositive neurons.

The expression of CRF in the paraventricular nucleus was shown after inhalation of EOAH (Figure 3A-D). Control mice exposed to the FST had significantly higher mean number of CRF-immunoreactive neurons than normal mice (Figure 3E). While the FST markedly increased the expression of CRF in the paraventricular nucleus in mice receiving inhalation of saline (*p* < 0.001), there was an increase in 5-HT expression in those receiving inhalation of EOAH (0.5 and 1.0 g). A one-way ANOVA revealed a significant main effect of the treatment on the expression of CRF-immunoreactive neurons in the paraventricular nucleus (F_3,31_ = 11.060, *p* < 0.001). Post hoc tests indicated that inhalation of EOAH (0.5 and 1.0 g) induced marked increases in the expression of CRF-immunoreactive neurons in the paraventricular nucleus compared with saline-treated mice (*p* < 0.05, *p* < 0.001, Figure 3E).

### TH-immunoreactive neurons in the locus coeruleus

**Figure 4:**
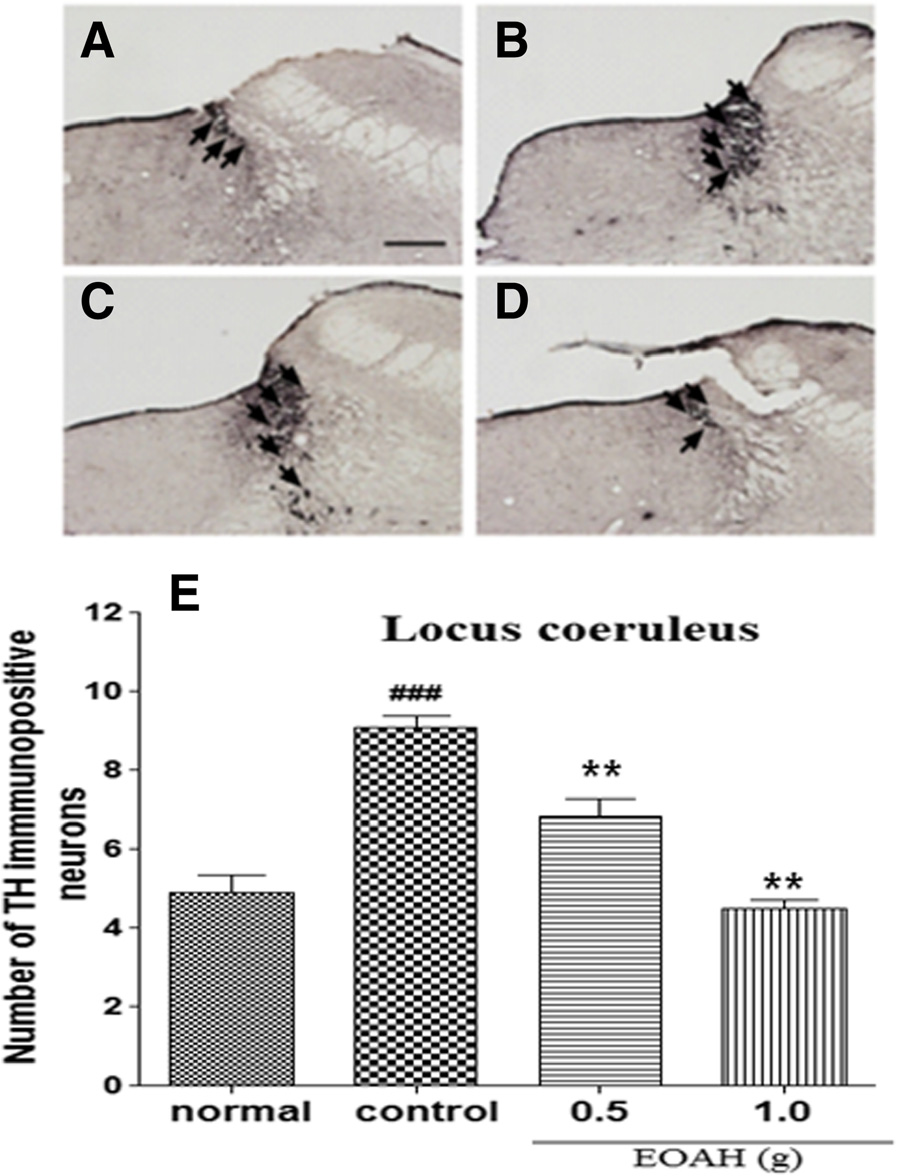
**Effect of inhalation of EOAH on TH expression in the locus coeruleus in mice subjected to the FST.** Photographs represent the distribution of TH-immunoreactive cells in the locus coerleus of normal (**A**, *n* = 8), control (**B**, *n* = 9), EOAH 0.5 g (**C**, *n* = 9) and EOAH 1.0 g (**D**, *n* = 9) groups. The number of the TH-immunostained neurons among the groups (**E**) was analyzed by one-way ANOVA *post-hoc* Newman-Keuls test. Each value represents the mean ± S.E.M. ^###^ *p* < 0.001 compared to the normal group, **p < 0.01 compared to the control group. Sections were cut coronally at 30 μm and the scale bar represents 200 μm. Arrowheads indicate TH immunopositive neurons.

The expression of TH in the paraventricular nucleus was shown after inhalation of EOAH (Figure 4A-D). Similar to the CRF data, mice exposed to the FST showed a significant difference in the mean number of TH-immunoreactive neurons in the locus coeruleus from normal group (*p* < 0.001, Figure 4E). However, inhalation of EOAH significantly reduced the expression of TH in the locus coeruleus compared to saline inhalation. A one-way ANOVA revealed a significant main effect of the treatment on the expression of TH-immunoreactive neurons in the LC (F_3,31_ = 36.040, *p* < 0.001). Post hoc tests indicated that inhalation of EOAH (0.5 and 1.0 g) induced marked increases in the expression of TH-immunoreactive neurons in the locus coeruleus compared with treatment of saline (*p* < 0.001, Figure 4E).

## Discussion

The present results demonstrated that the inhalation of EOAH effectively attenuated depression-like behavior, increases in brain expressions of CRF and TH, a decrease in brain expressions of 5-HT in response to 6 min of forced swimming or immobilization stress. Behavioral responses to the FST and TST are consistent with previous findings [3,4]. The FST and TST are useful tests for screening antidepressant drugs and exploring their mechanisms of action, because of its good reliability and predictive validity [21]. The FST and TST commonly trigger psychological despairs with no escapable hope from immobilization by learning that escape from the current situation is impossible. Thus, results in no hope or no trial to escape and consequently develop depression status [17].

Pharmacologically, a variety of anti-depressants and related-compounds with potential anti-depressant activity reduce the duration of immobility in the FST and TST [22,23]. Therefore, the reduction of immobility is the important factor to develop the pharmacological approaches to the treatment of depression-like behaviors in experimental designs. Our results suggest that inhalation of EOAH reduces depression-like behaviors in the FST and TST. However, our results showed that lower doses of EOAH did not produce a significant effect on depression-like behaviors in the FST. This differs with effects of EOAH at lower doses in the TST as demonstrated by a significant decrease in depression-like behaviors. Such inconsistencies may be at least in part related to differences in response to EOAH between the FST and TST. In support of this, one study has shown that TST is more responsive to neurochemical changes than the FST [24]. The present results are similar to others who demonstrated that pure fragrance of aroma oil and essential oil from plants decreased depression-like behaviors in the FST and TST [25,26]. Further support for a role of EOAH is the observation that systemic administration of methyl eugenol, which is known to be one of volatile components of *Asarum heterotropoides* [27], produced anti-anxiety and anti-depression effects in the FST [28].

Depression-like behaviors induced by stressors have been linked to endogenous systems of CRF and monoamines including 5-HT and NE [29,30]. Pharmacological studies have shown that CRF1 receptor antagonist, 5-HT1 receptor agonist, and adrenoreceptor antagonist can decrease depression-like behaviors, indicating that endogenous systems of CRF and monoamines including 5-HT and NE in the brain contribute to development of depression [31-34]. Thus, it is not unexpected that normalization of these systems attenuates depression-like behaviors [35-37]. In our study, mice subjected to forced swimming stress showed significant increases in brain expressions of CRF and NE, and significant decreases in brain expression of 5-HT. These results are consistent with previous findings, suggesting that endogenous systems of CRF, 5-HT, and NE in the brain plays an important role in modulating depression-like behaviors. For example, the expression of CRF mRNA or CRF immunoreactivity in the hypothalamic neurons is known to be up-regulated by stressor including forced swimming stress [38,39]. Similarly, CRF 2 mutant mice showed increased immobility time in forced swimming test [40]. It is noteworthy that one particular stressor, forced swimming, was associated with decreases of extracellular 5-HT in several brain regions including the dorsal raphe nucleus, lateral septum, and amygdala [41,42]. Also, serotonin receptor 1A knockout (KO) mice or serotonin transporter KO mice exhibited a decreased immobility time in the forced swim test [43,44]. In contrast, exposure to forced swimming considerably enhanced the release of norepinephrine in the locus coeruleus [45,46]. In addition, norepinephrine transporter KO mice have been shown to reduce immobility in the FST and TST [44]. Therefore, it is highly likely that depression-like behaviors are due to activation of CRF neurons in the hypothalamus and noradrenergic neurons in the locus coeruleus and suppression of serotonergic neurons in the dorsal raphe nucleus.

Most importantly, results showed that EOAH at the dose of 1.0 g significantly reduced the expected increases in the expression of CRF positive neurons in the paraventricular nucleus, the expected decreases in the expression of TH in the locus coeruleus and the 5-HT positive neurons in the dorsal raphe nucleus in mice subjected to forced swimming stress. Also, EOAH at the dose of 1.0 g reduced a significant increase in immobility time in the FST and TST. Based on a role for endogenous systems of CRF, 5-HT, and NE in the brain, one possible mechanism where EOAH could diminish depression-like behaviors is by affecting brain levels of 5-HT, CRF, and TH. Recently, it has been proposed a hypothetical model that might explain how inhalation of volatile essential oils activates brain areas [33]. In brief, volatile molecules of essential oil may diffuse into the systemic circulation through the lung, which inhalation takes them to. Subsequently, they are transported to brain areas. In addition, the molecules activate the olfactory system connected to the limbic system via binding to olfactory receptors. Determination of the specific mechanisms involved in anti-depressant effects induced by EOAH will require additional study.

## Conclusion

In conclusion, our results suggest that the reduced stress response produced by inhalation of EOAH is most likely mediated via an activation of serotonergic system and an inhibition of corticotropinergic and catecholaminergic system in the brain.

## Abbreviations

FST: Forced swimming test
TST: Tail suspension test
CRF: Corticotropin releasing factor
5-HT: Serotonin
TH: Tyrosine hydroxylase
